# Assessing the critical success factors for implementing industry 4.0 in the pharmaceutical industry: Implications for supply chain sustainability in emerging economies

**DOI:** 10.1371/journal.pone.0287149

**Published:** 2023-06-15

**Authors:** Binoy Debnath, Md Shihab Shakur, A. B. M. Mainul Bari, Joy Saha, Wazida Akter Porna, Mostarin Jahan Mishu, Abu Reza Md. Towfiqul Islam, Muhommad Azizur Rahman

**Affiliations:** 1 Department of Industrial and Production Engineering, Bangladesh University of Engineering and Technology, Dhaka, Bangladesh; 2 Department of Mechanical and Production Engineering, Ahsanullah University of Science and Technology, Dhaka, Bangladesh; 3 Department of Disaster Management, Begum Rokeya University, Rangpur, Bangladesh; King Khalid University, SAUDI ARABIA

## Abstract

The emerging technologies of Industry 4.0 (I4.0) are crucial to incorporating agility, sustainability, smartness, and competitiveness in the business model, enabling long-term sustainability practices in the pharmaceutical supply chain (PSC). By leveraging the latest technologies of I4.0, pharmaceutical companies can gain real-time visibility into their supply chain (SC) operations, allowing them to make data-driven decisions that improve SC performance, efficiency, resilience, and sustainability. However, to date, no research has examined the critical success factors (CSFs) that enable the pharmaceutical industry to adopt I4.0 successfully to enhance overall SC sustainability. This study, therefore, analyzed the potential CSFs for adopting I4.0 to increase all facets of sustainability in the PSC, especially from the perspective of an emerging economy like Bangladesh. Initially, sixteen CSFs were identified through a comprehensive literature review and expert validation. Later, the finalized CSFs were clustered into three relevant groups and analyzed using a Bayesian best-worst method (BWM)-based multi-criteria decision-making (MCDM) framework. The study findings revealed that "sufficient investment for technological advancement", "digitalized product monitoring and traceability", and "dedicated and robust research and development (R&D) team" are the top three CSFs to adopt I4.0 in the PSC. The study’s findings can aid industrial practitioners, managers, and policymakers in creating effective action plans for efficiently adopting I4.0 in PSC to avail of its competitive benefits and ensure a sustainable future for the pharmaceutical industry.

## 1. Introduction

A sustainable pharmaceutical supply chain (PSC) aims to provide an effective end-to-end information and material flow system to attain a responsive, agile, accurate, and data-driven supply chain (SC). In the current competitive market, the pharmaceutical industry needs to evolve into a lean, focused organization with massive research and innovation activities to generate sustainable income streams from developing specialized goods and technologies [1]. The convergence of Industry 4.0 (I4.0) and healthcare has been influencing the big pharmaceutical industries for the past few years, resulting in ’Pharma 4.0’, resulting from adopting I4.0 concepts to the pharmaceutical and life sciences sectors [2]. The principal drivers of I4.0 in the convergence process include cloud computing, augmented reality (AR), virtual reality (VR), the Internet of Things (IoT), cyber-physical systems (CPS), big data analytics (BDA), cybersecurity, simulation, autonomous robots, etc., [3]. These emerging technologies are transitioning the pharmaceutical industry toward modern-day digital and technology-dependent manufacturing systems [4]. This change assists businesses in leveraging technology to reach their objectives by operating more efficiently at a lower cost but in a competitive and agile manner [5]. Even though I4.0 originated in manufacturing, it has significantly impacted strategic SC operations [6].

I4.0 technologies are attracting the pharmaceutical industry for several reasons. In the post-COVID-19 period, the need for achieving sustainability in the SC has intensified in various sectors [7]. Achieving sustainability will improve various aspects of PSC, such as customer satisfaction level, responsiveness, flexibility, communication, service fill rate, etc. Moreover, maintaining sustainable accessibility and affordability of indispensable medicines and treatments is critical. Therefore, disrupting the PSC might endanger human life, causing difficulties in healthcare operations and medication availability [8]. In such scenarios, emerging technologies of I4.0 can assist pharmaceutical industries in overcoming the issues hindering sustainability in this sector since adopting I4.0 facilitates businesses to attain the triple bottom line of sustainability, promoting economic progress, social well-being, and environmental conservation [9].

The instrumental role of I4.0 can enhance visibility and collaboration and build trust among all the SC stakeholders by changing the pharmaceutical business dynamics [10]. I4.0 offers a consolidated environment for PSC stakeholders for smooth information sharing, reducing the bullwhip effect in the SC, and increasing visibility to respond to disruptions [11]. Moreover, I4.0 enables long-term value creation and leads to a more agile, intelligent, and customized pharmaceutical industry, allowing pharmaceutical firms to gain a competitive edge [12]. Real-time monitoring and assessment of the whole SC, from raw materials to the finished product, are facilitated by the incorporation of the Internet of Things (IoT), artificial intelligence (AI), and blockchain, which offer data-driven views for formulating sustainable decisions [13]. To better understand the data provided, pharmaceutical businesses must integrate data-driven analytical tools like simulation, predictive analysis, data analytics, and optimization. These tools facilitate value creation for sustainable PSC management to obtain competitive advantages in sustainable practices [14]. Therefore, stakeholders must be aware of the latest technologies to ensure they can all function together to create a trustworthy data-driven value chain throughout the pharmaceutical industry [15]. Moreover, employees must be trained, and a culture transformation must occur to effectively adopt this awareness of the goals for an efficient transition to I4.0 [16].

Several studies have been conducted to integrate I4.0 into the pharmaceutical manufacturing industry. For instance, Arden et al. [17] initially identified various known or underlying risks related to specific new techniques and inconsistencies with current strategies for legislative compliance in order to create regulatory structures that promote I4.0 in the pharmaceutical business. However, the study hasn’t mentioned how the regulatory scheme incentivizes investment in the pharmaceutical industry. Chen et al. [18] provided a comprehensive assessment of the vital elements of Pharma 4.0 concerning the regulatory driving factors for smart manufacturing, difficulties, and potential future advances. However, no quantitative or qualitative analysis was used to prioritize or develop a relationship-building framework to evaluate those vital elements. Reinhardt et al. [19] recognized and discussed the challenges through a survey regarding the holistic integration of I4.0 into the organizational culture of the pharmaceutical business. However, this research has advised the regulatory body, experts, and policymakers to work together but has not provided any action plans or strategies from the decision-makers’ perspective to incorporate I4.0 into the PSC in emerging business circumstances. Therefore, it is apparent that there exists a significant research gap in the literature on the pharmaceutical industry, particularly from the perspective of an emerging country like Bangladesh, to carry out an in-depth analysis of the critical success factors (CSFs) that significantly influence the I4.0 deployment to develop a sustainable SC. This gap in the literature is worth exploring to facilitate sustainable development in the pharmaceutical industry. This research, thereby, aims to address this gap by answering the following significant research questions (**RQs**):

***RQ1*:** What significant CSFs influence I4.0 deployment in the pharmaceutical industry in an emerging economy?
***RQ2*:** How can these CSFs be investigated hierarchically from a sustainable PSC perspective?
***RQ3*:** What values do these CSFs provide to the pharmaceutical SC towards achieving the SDGs?
To address these RQs, the subsequent research objectives (**ROs**) need to be achieved:
***RO1*:** To determine the significant CSFs of I4.0 implementation toward sustainable development in the PSC.
***RO2*:** To analyze and prioritize the identified CSFs.
***RO3*:** To equip SC managers, practitioners, and policymakers with important insights and knowledge that will help them effectively implement I4.0 throughout the PSC and meet SDGs.

A Bayesian Best Worst Method (BWM) framework has been proposed to accomplish the **RO**s of this study. The Bayesian BWM is a robust technique for generating multi-criteria decisions and determining criteria weight factors [20]. In many real-world multi-criteria evaluation problems, where numerous criteria have the same effect on decision-making, information gaps might exist after aggregating the individual compared pairings [21]. In such cases, the Bayesian BWM entails selecting one best and one worst criterion from a collection of observed criteria and overcoming the information gap problem by functioning as a group within the overall cluster. There are significant advantages to using Bayesian BWM for CSFs prioritization over other distinct pairwise comparison-based MCDM methods, like the Analytic Network Process (ANP) and the Analytic Hierarchical Process (AHP). Bayesian BWM generates a more reliable expert opinion matrix and requires fewer pairwise comparisons than AHP and ANP [22]. Again, while the conventional linear BWM depends on the opinion of an individual expert to establish the weights of various criteria [23], Bayesian BWM employs a statistical estimation-based method to allow collective or group decision-making without information loss [24]. Thus, Bayesian BWM has overcome the limitations of classic linear BWM by combining feedback data from multiple experts to provide a set of optimal group weights for more accurate evaluation [25]. Moreover, Bayesian BWM decreases the potential of making a mistake while comparing pairs of criteria, increasing the dependability of the findings [26].

### 1.1 Motivation, research gap, and study contributions

The pharmaceutical industry is undergoing continuous pressure to embrace sustainable practices and mitigate their adverse environmental impact [27]. Adopting I4.0 technology can provide a possible remedy to this issue [28]. It facilitates reducing material waste throughout the product life cycle, allowing more environmentally friendly and energy-efficient manufacturing. Again, the PSC and the pharmaceutical production process can be significantly benefitted from incorporating digitalization, autonomous systems, computers, and I4.0 technology [29, 30]. I4.0 connects all the SC operations and shares real-time data, enhancing the logistical processes [31]. By enabling the sharing of data and insights to improve the SC, I4.0 technology facilitates collaboration among the SC stakeholders.

I4.0 technology applies automation, intelligent machines, and data processing advancements and can potentially transform the infrastructure for pharmaceutical manufacturing and distribution completely [32, 33]. Moreover, the application of I4.0 can also be leveraged in the pharmaceutical industry to improve personalized medication enabling the mass customization of products with increased flexibility and efficiency of the manufacturing processes [34]. Through AI and data analytics, pharmaceutical companies may develop specialized medications for each patient, minimizing waste and enhancing patient care. The predictive maintenance technology of I4.0 can track the condition of production equipment and foresee when the repair is necessary, lowering the likelihood of substitution and, thus, saving money and reducing the waste of resources. Furthermore, while I4.0 technologies are quickly developing, their application in PSC remains fragmented [35]. I4.0 technologies can play an important role in PSC to facilitate sustainability and enhance the quality of human lives. Such possibilities have motivated this study to thoroughly examine PSC for efficient adoption of I4.0 while improving the sustainability aspects (social, environmental, and economic).

However, few studies have been conducted to analyze the CSFs to implement I4.0 in manufacturing businesses of distinctive areas. For example, Pozzi et al. [36] used an iterative approach for exploring the CSFs to implement I4.0 in improving the manufacturing performance of eight industries, except for the pharmaceutical sector. Bhatia & Kumar [37] have empirically examined the CSFs for adopting I4.0 technologies in the Indian car manufacturing sector. Khin and Kee [38] proposed a triadic conceptual model to explore the elements impacting manufacturing companies’ decisions to embrace I4.0, particularly in SMEs. Kumar et al. [39] used the fuzzy DEMATEL approach to identify CSFs to implement I4.0 in a circular SC. Nwaiwu et al. [40] explored the CSFs to integrate I4.0 in the small medium enterprises (SME) manufacturing industry employing a mixed quantitative and qualitative method. However, these prior studies have a different focus than the pharmaceutical business utilizing older methodologies owing to several drawbacks that can be addressed using a newer and more effective tool, such as the Bayesian BWM.

AI, blockchain, IoT, BDA and data integration, CPS, VR and augmented reality, robotics and automation, and advanced analytics are the most explored I4.0 technologies in the pharmaceutical sector, which can increase operational effectiveness, save costs, and improve product quality [41]. Kulkov [42] has emphasized the use of AI in pharmaceutical business analytics, master data management, additional research and development (R&D) projects, and human resource analysis and reporting. However, the work has not mentioned how AI applications can adapt in different circumstances to achieve integration goals in the pharmaceutical business. Moreover, Kumar et al. [43] examined the importance of data analytics, AI, and other new technologies in the pharmaceutical industry’s continuous manufacturing process for SC security and improved product safety. However, the research has not addressed how much transparency the continuous manufacturing process may bring to PSC or how this can contribute to sustainability.

Again, Haji et al. [44] have examined the CSFs for establishing a safe PSC and found cooperation between the organizations, support from the government, quality of information, integration, and interoperability to prevent counterfeit risks, and several PSC phases are integrating traceability technologies. Therefore, various stages of value chains are influenced by I4.0’s enhanced traceability of products and materials, enhanced vendor performance because of real-time data transmission and coordination with suppliers, and intelligent logistics and vehicle routing systems that contribute to more accurate forecasting and planning [45]. However, so far, no previous studies have examined long-term practices associated with I4.0 adoption in PSC sustainability. Moreover, no earlier study has investigated such aspects using a multi-criteria decision-making (MCDM) framework from an emerging economy perspective. Therefore, the novelty of this proposed research lies in the amalgamation of the concepts of Industry 4.0, sustainability, emerging economy, and Bayesian BWM-based MCDM framework with the pharmaceutical industry, which has not been tried in any previous research. This study intends to fill the existing gap in the literature by making the following research contributions:

1. To identify the CSFs to implement I4.0 in the pharmaceutical sector of a developing economy like Bangladesh.
2. To establish the connection between I4.0 technologies and PSC sustainability.
3. To propose a Bayesian BWM-based framework to prioritize the identified CSFs and analyze their influences.
4. To provide managers, industry leaders, and policymakers of emerging economies with important insights to make strategic decisions to implement I4.0 in the pharmaceutical industry while ensuring the sustainable development of this sector.

The remaining article has been arranged into the following sections: Section 2 presents the research methodology, including the study context and data collection process. The results are presented in section 3. Section 4 discusses the study’s results and the theoretical, managerial, policy, and sustainability implications. Section 5 finally concludes the study and offers some suggestions for future research.

## 2. Methodology

### 2.1 Study context

The pharmaceutical business is one of Bangladesh’s fastest-growing industries. Bangladesh’s pharmaceutical sector is regarded as the most robust among 48 emerging economies for producing generic life-saving drugs [46]. With an increasing number of businesses permitted to export medicines to developed nations, the pharmaceutical sector of Bangladesh has established itself as a major worldwide supplier of generic medications [47]. Now, the pharmaceutical sector of Bangladesh meets 97% of its overall medicinal demand and exports worldwide due to its low-cost labor market [48].

The pharmaceutical business is unquestionably one of Bangladesh’s most significant and highly dynamic industrial sectors. Over 300 pharmaceutical manufacturing companies are operating in Bangladesh, exporting to 125 countries all over the globe in 2021, earning more than USD 100 million annually [49]. After the RMG industry, it has the highest exports in the country [50]. With the excellent quality and cheap cost of Bangladeshi pharmaceutical products and their competitive packaging and presentation, there is an incredible demand for them in the global market [51]. The sector has risen to be the second-largest potential exporter of medicines worldwide [52]. Bangladesh aims to establish a strong foothold in the worldwide pharmaceutical industry by having 30 top-tier medicine manufacturers by the end of the next ten years [53]. Bangladesh’s pharmaceutical market is increasing by 114% over current levels and is estimated to exceed $6 billion by 2025 [54]. Despite being the country’s most sophisticated and modern industrial sector, the industry is still regressive regarding implementing I4.0 to facilitate sustainability. This lag has immense negative implications regarding production, safety stock, inventory of raw materials, energy consumption, chemical pollution, and longer medicine shelf-life. Motivated by this situation, this study has been conducted, focusing extensively on the pharmaceutical industry of Bangladesh.

### 2.2 Data collection

The study proposed a Bayesian BWM approach to examine the CSFs to incorporate I4.0 in PSC while promoting sustainability. The data collection and survey have been conducted in two phases. We initially developed a listing of the sixteen most important CSFs for I4.0 deployment in the pharmaceutical business in emerging nations after conducting an extensive analysis of the related studies available in the Google Scholar and Scopus databases, published from 2018 to 2022. The key phrases applied to explore the CSFs from the literature included the following: "CSFs for the sustainable supply chain in the pharmaceutical industry" OR "CSFs for Industry 4.0 implementation in the pharmaceutical sector" OR "critical factors to integrating industry 4.0 in the sustainable supply chain" OR "factors to achieve sustainability with industry 4.0 implementation in the pharmaceutical supply chain", and so on. The sixteen initially identified CSFs were then sent to the participating experts as a Google Forms survey to authenticate and cluster the CSFs for subsequent assessment. Here, a non-random sampling method called purposive sampling was employed to pick industry practitioners [55, 56] to take part in the survey, which is critical for gathering enough responses in a specific category [57, 58]. Purposive sampling involves a professional appraisal of the sample’s suitability that makes a persuasive case that a particular set of respondents provides a more trustworthy foundation for experimental survey findings than other sampling methods [59, 60]. Experts were chosen based on at least seven years of relevant employment experience in the pharmaceutical business, healthcare, or academia, enough pharmaceutical operation and research knowledge, a deep understanding of I4.0 technologies, PSC, etc. Most of the industry experts were SC professionals, practitioners in R&D, and technological consultants. Table 1 summarizes the characteristics of participating experts. The chosen respondents are actively involved in the pharmaceutical sector in some way, with extensive knowledge and experience in SCM, planning, logistics, marketing, research and development, information and technology, procurement, distribution, administration, and other related sectors. These standard selection criteria have been preserved to ensure that the expert’s opinion is more relevant while avoiding significant subjective bias [61, 62].

**Table 1:** A summary of the experts’ profile.

Expert No	Expertise field	Educational Qualification	Experience (Years)	Validate and cluster CSFs	Ranking of CSFs
1	Planning	M. Engg in Advanced Engineering Management	12	✓	✓
2	Supply chain	Masters in Supply chain management and logistics	18	✓	✓
3	Procurements	Masters in Procurement and Supply Management	15	✓	✓
4	Administration	M. Engg in Advanced Engineering Management	10	✓	⨉
5	Distribution	M. Engg in Advanced Engineering Management	8	✓	⨉
6	Marketing	MBA in Marketing	9	✓	✓
7	Information and technology	M.Sc in Computer Science	4	✓	✓
8	Information and technology	M.Sc in Electrical and Communication Engineering	6	✓	✓
9	Administration and Logistics	M.Sc in Supply chain management and logistics	8	✓	✓
10	Logistics	Masters in logistics Management	5	✓	⨉
11	Supply chain	M.Sc in Supply Chain Management	12	✓	✓
12	Planning	M. Engg. in Advanced Engineering Management	16	✓	⨉
13	Administration	M. Engg. in Advanced Engineering Management	7	✓	✓
14	Distribution	B.Sc in Industrial and Production Engineering	10	✓	✓
15	Research and Development (R&D)	Ph.D. in Industrial and Production Engineering	7	✓	✓
16	Supply chain	MBA in Supply chain management	5	✓	✓

The survey questionnaire provided to the experts to verify the primarily identified CSFs, is provided in **Appendix A** of the S1 File. The expert’s answers were received in a ’yes’ and ’no’ format regarding the validation of the relevance of the corresponding CSF. After getting the feedback, the ’yes’ response was considered ’1’, and the ’no’ was considered ’0’. The arithmetic mean was then calculated for each CSFs, and the CSFs feedback mean score greater than 0.7 were selected for further evaluation.

The experts eliminated two CSFs ("Organizational culture" and "Technical Capabilities") and added one additional driver ("Dedicated and robust Research and Development (R&D) team") by considering the relevance to the study. The finally selected list of sixteen CSFs is provided in Table 2. **Table A1** of **Appendix A** in the S1 File briefly describes the finalized CSFs.

**Table 2:** Finalized CSFs list to implement I4.0 toward developing sustainable PSC.

SL No	CSF	Source
1	Existent sustainable organizational strategies	[63, 64]
2	Auspicious government policies and legislation	[65]
3	Sufficient investment in technological advancement	[66]
4	Establishment of a robust technical support team	[67]
5	Training programs for employee skill advancement	[9]
6	Integration of advanced database management system	[68]
7	Proper support from top management	[69]
8	Incorporation of emerging sustainable technologies	[13, 70]
9	Establishment of the computer-integrated cloud manufacturing system	[71]
10	Dedicated and robust Research and Development (R&D) team	Expert Feedback
11	Secured and stable data communication network	[15]
12	Maintain proper collaborative communication among supply chain entities	[72]
13	Digitalized product monitoring and traceability	[73, 74]; Expert Feedback
14	Decentralized management and flexible operations	[75]
15	Sourcing and application of smart and advanced equipment	[17]
16	Increased automation in material handling and inventory management	[76]

Following their identification, the CSFs were validated and classified into three major clusters- organization and government, technology and innovation, and information and networks. Twenty-five experts were invited to participate in the CSF validation and clustering survey, among which 16 responded (64% response rate). Table 3 outlines the main clusters and their corresponding CSFs.

**Table 3:** The major clusters and their corresponding CSFs.

Cluster Name	CSFs	Code
Organization and government-related cluster	Establishment of a robust technical support team	O1
	Existent sustainable organizational strategies	O2
	Auspicious government policies and legislations	O3
	Proper support from top management	O4
	Dedicated and robust Research and Development (R&D) team	O5
Technology and innovation-related cluster	Sufficient investment in technological advancement	T1
	Training programs for employee skill advancement	T2
	Sourcing and application of smart and advanced equipment	T3
	Incorporation of emerging sustainable technologies	T4
	Increased automation in material handling and inventory management	T5
	Decentralized management and flexible operations	T6
Information and network-related cluster	Secured and stable data communication network	I1
	Integration of advanced database management system	I2
	Establishment of the computer-integrated cloud manufacturing system	I3
	Digitalized product monitoring and traceability	I4
	Maintain proper collaborative communication among supply chain entities	I5

**Fig 1:**
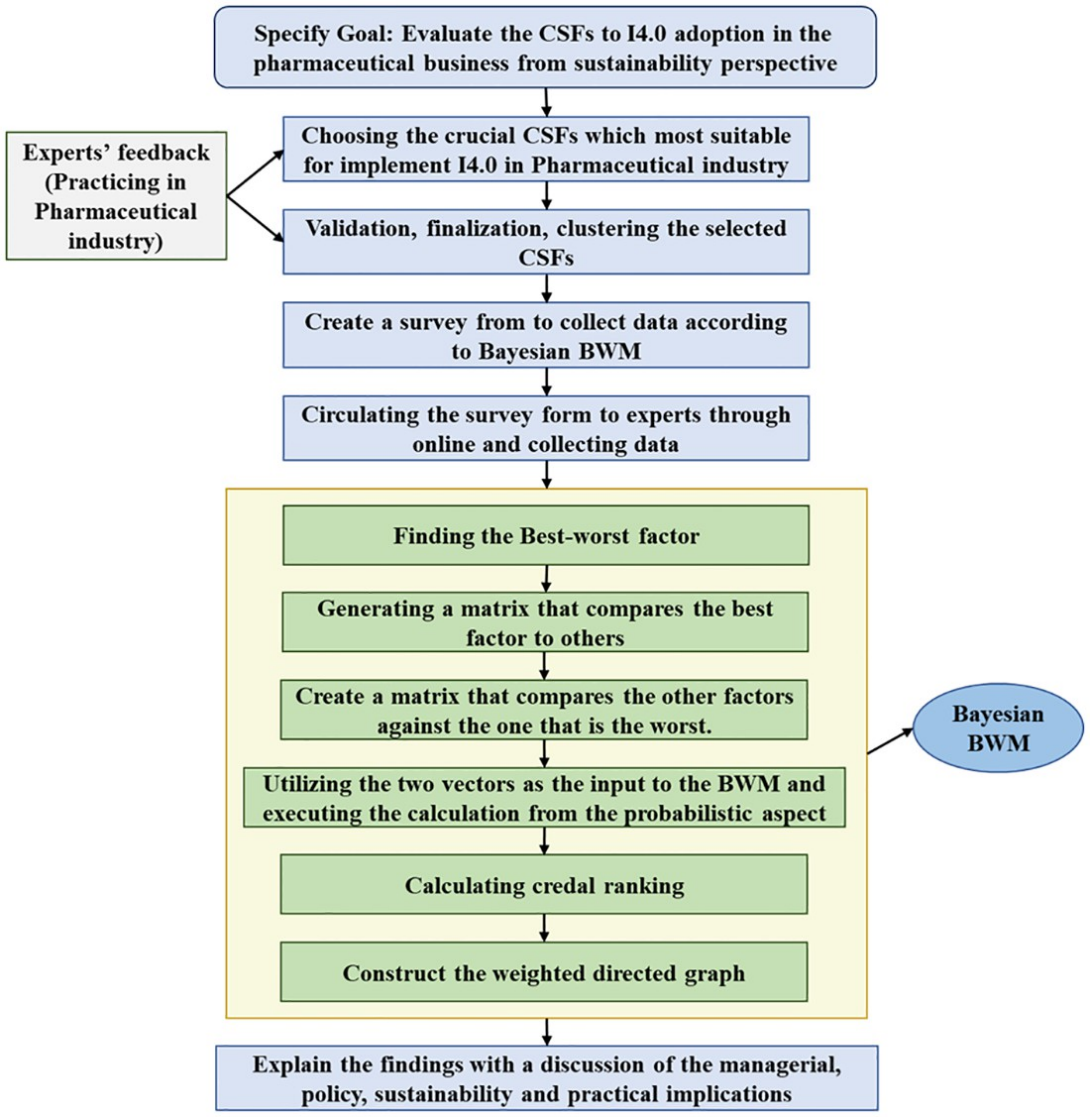
A proposed framework for the Bayesian BWM approach.

The experts were contacted again for the BWM survey in the second phase. The "Best-to-Others" and "Others-to-Worst" matrixes were generated from this survey data. Sixteen experts from the CSF validation phase were invited again to participate in the Bayesian BWM analysis survey [26, 77], among which 12 responded (75% response rate). Both surveys were conducted online using Google Forms for the convenience of the experts. The methodological framework of the study is represented in Fig 1.

### 2.3 Bayesian Best Worst method (BWM)

The Best Worst Method (BWM) is highly effective in making pairwise comparisons because it significantly minimizes the level of inconsistency present in the comparison data [78, 79]. Bayesian BWM is an upgraded form of the BWM method, a group decision-making technique for integrating the opinions of several experts in a probabilistic manner. The procedure is stochastic since pairwise comparisons are performed using the multinomial distribution, and global weights are computed using the Dirichlet distribution [80]. The salient advantage of Bayesian BWM is that it reduces the inconstancy in the comparison data more than the conventional BWM. In recent years, the Bayesian BWM approach has been applied in studies performed in a variety of sectors, as shown in **Table B1** of **Appendix B** in *supplementary materials*.

Let, a group of criteria, *G* = {*G*_1_, *G*_2_, … … …, *G*_*s*_} that are evaluated by several *m* specialists. The Bayesian BWM is implemented using the subsequent steps [81]:

1. **Selection of best and worst criterion:** The best ${(G}_{B}^{m})$ and worst $\left(G_{W}^{m}\right)$ criteria from the criteria set *G*. Each expert, *m*, is required to choose only one from the best and only one from the worst criteria. The experts just decide the best and worst criterion instead of making any pairwise comparisons in this step. The best criterion has the most importance, while the worst criterion has the lowest importance, according to the group *o*f specialists *m*. However, different experts’ choices could be distinct from each other’s.
2. **Developing the best to others pairwise comparison**: Expert *m* creates a pairwise comparison matrix of the best ${(G}_{B}^{m})$ criteria in the set of *G* and other criteria. Here, every expert chooses a number between one and nine to express their priority for presenting their choice of the best criteria over other criteria in *G*, as determined in step 1. Nine means that ${(G}_{B}^{m})$ is much more important than the other criteria, while one implies that the criteria are equally important. The collection of pairwise comparisons of expert *m* results in the vector "Best-to-Others," which is denoted by $X_{B}^{m}$ as follows:
$$X_{B}^{m}={(x}_{B1}^{m},x_{B2}^{m},\ldots \ldots \ldots ,x_{Bm}^{m});m=1,2,3,\ldots \ldots ,M$$ (1)
  where $x_{Bj}^{m}$ indicates the preferred criteria of expert *m*’s for the best criteria ${(G}_{B}^{m})$ over *g*_*j*_ ∈ *G*.
  The "Best-to-Others" vectors for every expert in this study’s clusters and sub-clusters have been shown in **Table C1-C4, Appendix C** of S1 File.
3. **Forming the others to worst pairwise comparison:** Similarly, *m* experts create a pairwise comparison matrix among the worst $\left(G_{W}^{m}\right)$ and other criteria for the set of *G*. The collection of pairwise comparisons of expert *m* results in the vector "Others-to-Worst," which is represented by $X_{W}^{m}$ as follows.
$$X_{W}^{m}={{(x}_{1W}^{m},x_{2W}^{m},\ldots \ldots \ldots ,x_{mW}^{m})}^{T}$$ (2)
  Where, $x_{1W}^{m}$ denotes the preference of an expert *m* for *g*_*j*_ ∈ *G* over the worst criteria $\left(G_{W}^{m}\right)$.
  All experts’ "Others-to-Worst" vectors for every cluster and their corresponding sub-cluster in this research have been shown in **Table D1-D4, Appendix D** of S1 File.
4. **Computation of aggregated weight**: The aggregated weights $\left(y^{*}=y_{1}^{*},y_{2}^{*},\ldots \ldots ..y_{p}^{*}\right)$ of all m experts and the individual expert’s weight (*y*^*m*^), are calculated with the following derived Bayesian BWM probabilistic technique, *where m* = 1, 2, ….. *M*; for each of the experts.
$$\left.X_{B}^{m}\right|y^{m}∼multinomial\left(1/y^{m}\right),\forall m=1,2,\ldots ..M,$$ (3)
$$\left.X_{B}^{m}\right|y^{m}∼multinomial\left(y^{m}\right),\forall m=1,2,\ldots ..,M,$$ (4)
$$\left.y^{m}\right|y^{*}∼Dir\left(\gamma *y^{*}\right),\forall m=1,2,\ldots ..,M,$$ (5)
$$\gamma \sim gamma\left(0.1,0.1,0.1\right)$$ (6)
$$y^{*}\sim Dir\left(1\right)$$ (7)

Where *Dir* is a Dirichlet distribution, *multinomial* is a multinomial distribution, and *gamma* (0.1, 0.1) represents gamma distribution with form parameters of 0.1. The probabilistic model is specified by Eq (7) without a closed-form solution.

Once the issue has been resolved using the Bayesian BWM, *S* samples are generated using the posterior distributions of the aggregated weight (y*). Depending on the aggregated weight, it is possible to assess the importance of each criterion and the extent to which one criterion is preferred over another when considering all experts’ opinions.

#### 2.3.1 Group decision-making

In this study, the expert’s opinions are processed using a group decision-making method (Munim et al., 2022 [81]). This group decision-making method utilizes a joint probability distribution approach. Here, the *m*^th^ decision makers (DMs), *m* = 1, ….., *M*, evaluate the set of criteria *G* = {*G*_1_, *G*_2_, … … …, *G*_*s*_} by providing a vector ${(G}_{B}^{m})$ and $\left(G_{W}^{m}\right)$. Let the total of all vectors in the base be indicated as superscript ^1:*M*^ turning the best criteria as ${(G}_{B}^{1:M})$ and worst criteria $\left(G_{W}^{1:M}\right)$. The overall optimal weight is *y*^*agg*^. The *y*^*agg*^ estimation is related to some secondary variables. The optimal weight of *m* DMs is computed by *y*^*m*^, *m* = 1, ….., *M*. Thus, the Bayesian-BWM model computes *y*^*agg*^ and *y*^1:*M*^ simultaneously before doing any statistical calculation. Given the provided data, it is necessary to write the joint probability distribution of all random variables. While doing group decision-making in BWM, $G_{B}^{1:M}$ and $G_{W}^{1:M}$ are given, both *y*^*agg*^ and *y*^1:*M*^ need to be calculated accordingly. Therefore, the joint probability can be formulated as

$$P\left(y^{agg},y^{1:M}|G_{B}^{1:M},G_{W}^{1:M}\right)$$ (8)

The individual variable probability can be computed using the probability rule by following Eq 9 when Eq 8 calculates the probability.


$$P\left(u\right)=\sum_{y}P\left(u,v\right)$$ (9)

Here, u and v are two arbitrary random variables.

## 3. Results

This section presents the rankings of CSFs obtained using the Bayesian BWM approach. For every cluster, a weighted directional graph was generated with the vertices representing CSFs and corresponding weight, which was determined by the average of the aggregated weight (y*). Every line $M\overset{p}{\to }N$ denotes *M* is more important than *N*, owing a confidence level of ’*p*’. In essence, the graph’s overall structure encompasses the hierarchy of a set of criteria in terms of credal ranking, with each line symbolizing a unique credal ordering.

**Fig 2:**
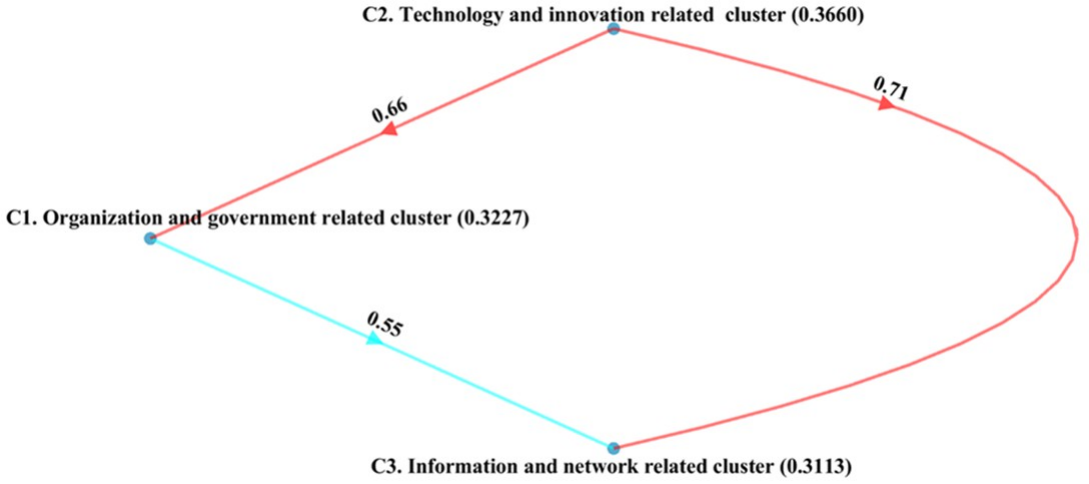
Ranking of main clusters.

Fig 2 shows the main three clusters with their credal ranking of technology and innovation-related cluster (0.3660), organization and government-related cluster (0.3227), and information and network-related cluster (0.3113). The technology and innovation-related cluster is the most significant as it is comparatively weighted higher than the information and network-related and technology and innovation-related clusters. Therefore, management must pay more attention to the CSFs relating to the technology and innovation-related cluster. However, organization and government-related and information and network-related clusters weigh close to each other. The technology and innovation-related cluster was revealed to be more important than the organization and government-related cluster (with a 0.66 confidence score) and the information and network-related cluster (with 0.71 confidence). Likewise, organization and government-related cluster was more implemented than information and network related, with a 0.55 confidence score.

**Fig 3:**
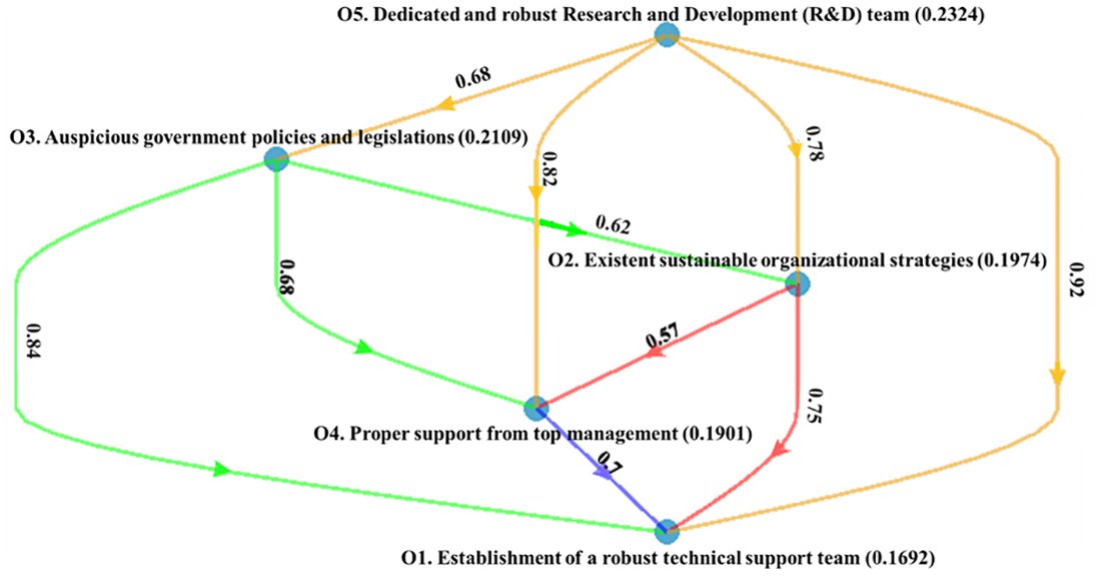
Ranking of organization and government-related CSFs.

Fig 3 illustrates the local credal hierarchy of CSFs within the organization and government-related cluster. The dedicated and robust Research and Development (R&D) team (0.2324) is found to be the most significant CSF, followed by auspicious government policies and legislations (0.2109), existent sustainable organizational strategies (0.1974), proper support from top management (0.1901), and Establishment of a robust technical support team (0.1692) respectively. Among the CSFs of the organizational and governmental-related cluster, dedicated and robust Research and Development (R&D) team weighed higher. Therefore, a dedicated and robust Research and Development (R&D) team is required to set the strategies, action plan, and policies to adopt I4.0 for sustainability in PSC. Dedicated and robust Research and Development (R&D) is involved in all the organizational and governmental operation phases to achieve sustainability in the PSC. The pharmaceutical business may contribute to ensuring that necessary pharmaceuticals and treatments are accessible to people worldwide by investing in R&D, addressing probable risks, and supporting responsible and ethical activity in the SC. Moreover, government policy and regulations may be quite effective at encouraging and fostering sustainable practices in the pharmaceutical sector, eventually resulting in a more environmentally friendly and resilient SC.

**Fig 4:**
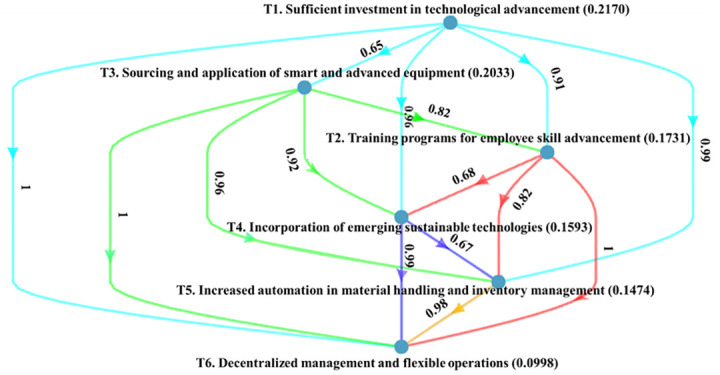
Ranking of technology and innovation-related CSFs.

Fig 4 represents the local credal hierarchy of the CSFs within the technology and innovation-related cluster. Sufficient investment in technological advancement (0.2170) is found to be the most significant CSF, followed by sourcing and application of smart and advanced equipment (0.2033), training programs for employee skill advancement (0.1731), incorporation of emerging sustainable technologies (0.1593), increased automation in material handling and inventory management (0.1474), and decentralized management and flexible operations (0.0998) respectively. For implementing I4.0 technologies, the pharmaceutical industry first needs sufficient investment in technological advancement in the emerging economy context. Once the pharmaceutical industry can get enough investment, they can go for various technologies and innovations. Therefore, sufficient investment in technological advancement is fundamental for achieving technological and innovation-related advancement. To promote innovation, growth, sustainability, and positive change and to maintain competitiveness in their SC operations, businesses must invest in technological advancement.

**Fig 5:**
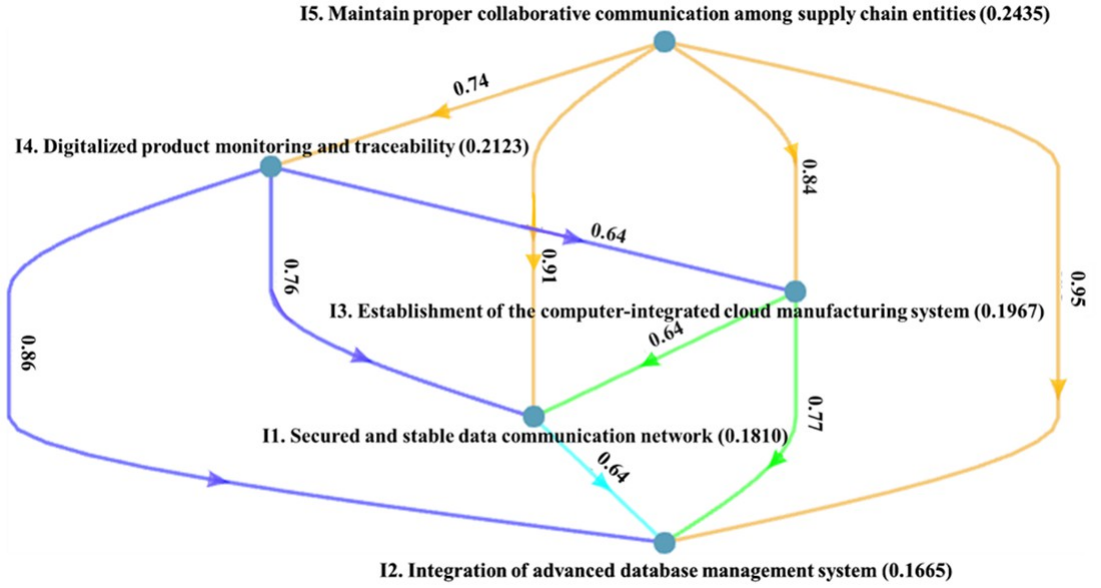
Ranking of information and network-related CSFs.

Fig 5 depicts the local credal hierarchy of the CSFs regarding the information and network-related cluster. Maintaining proper collaborative communication among supply chain entities (0.2435) is identified to be the top three CSFs in information and network-related cluster, followed by Digitalized product monitoring and traceability (0.2123) and Establishment of the computer-integrated cloud manufacturing system (0.1967). Maintaining proper collaborative communication among supply chain entities (0.2435) weighs higher than all the CSFs in information and network-related cluster. I4.0 significantly emphasizes the fusion of digital and physical systems, necessitating intense coordination and communication amongst many SC entities. Moreover, maintaining proper collaborative communication among SC entities creates a competitive advantage by enhancing customer service quality, profit generation, resource utilization, and cost reduction by promoting communication among SC partners to increase sustainability.

**Fig 6:**
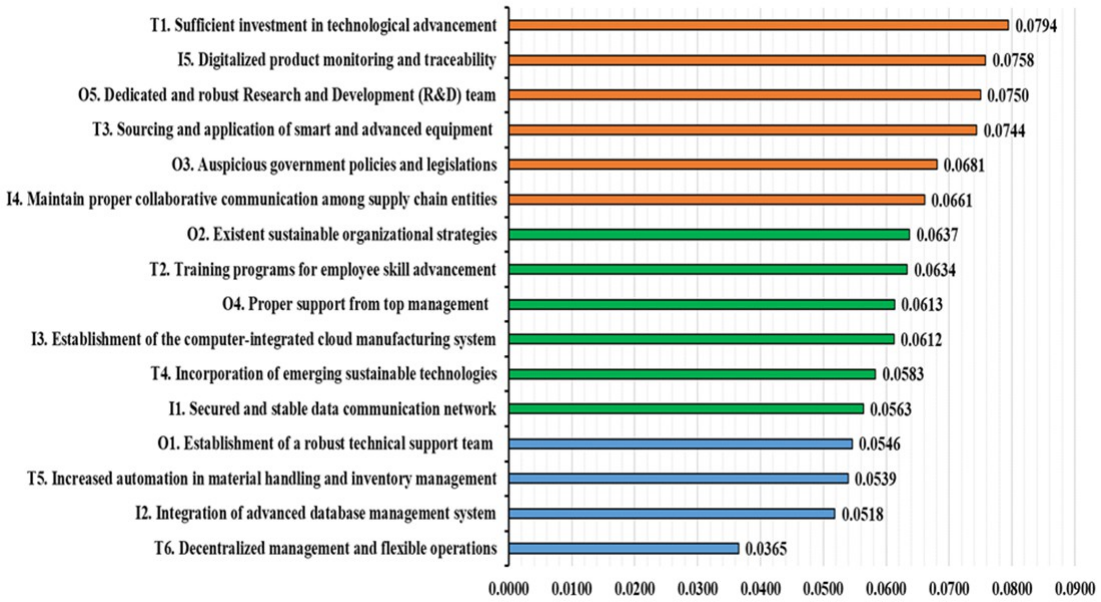
Global ranking of all significant CSFs.

Fig 6 illustrates the global rankings of all CSFs. The global hierarchy of all CSFs is calculated by multiplying weights at the cluster level with the local weights of every CSF. The study outcome indicates that sufficient investment in technological advancement (0.0794) is the most significant CSF, and decentralized management and flexible operations (0.0365) is the least significant CSF. The confidence score matrices for all clusters are provided in **Tables E1-E4**, **Appendix E** of the S1 File.

## 4. Discussion

Fig 6 shows the proposed structured framework with Bayesian BWM is used to rank the CSFs for implementing I4.0 in the PSC Sustainability context of an emerging economy. “Sufficient investment in technological advancement (T1)” was ranked top and determined to have the most influential role in implementing I4.0 in the pharmaceutical industry toward sustainable SC development because sufficient investment enables stakeholders to increase tangible and intangible assets to strengthen the pharmaceutical industry’s infrastructures of I4.0 to remain competitive in the rapidly evolving digital economy. Moreover, the technological investment will assist PSC in applying sustainable development tools to achieve technological changes such as recycling, waste minimization, substituting non-sustainable materials, changing production processes, pollution control, and more efficient resource utilization. Therefore, pharmaceutical industries should emphasize acquiring substantial investment for technological advancements to ensure growth and facilitate the holistic transformation of the SC towards technical sustainability. Arden et al. [17] also discovered the relevance of investment for the technological advancement of I4.0 in the pharmaceutical industry on SC performance, resilience, agility, flexibility, and visibility.

“Digitalized product monitoring and traceability (I5)” is found to be the second most important CSF in implementing I4.0 in PSC sustainability. The pharmaceutical industry deals with various reagents, solvents, buffers, leaves, fungi, and other materials that produce various vital products: medicine, antibiotics, vitamins, critical drugs, and vaccines. In such settings, dealing with various medications in their short life cycle becomes difficult without effective technology management. I4.0 implementation involves adopting various recent digital technologies, such as blockchain, IoT, CPS, and robotics, which facilitate the monitoring and traceability of pharmaceutical products so that they are notcompromised during transportation and storage. Throughout the production and SC cycle, digital transformation of product monitoring and traceability can reshape whole sectors, redefine established business models, and improve consumer experiences, allowing industries to be sustainable in the long term. Previously, Leal et al. [82] also found the importance of adigitalized product monitoring system in overcoming PSC hurdles and advised using advanced quantitative analysis.

“Dedicated and robust Research and Development (R&D) team (O5)” is prioritized as the third most important CSF because it not only improves the health of the population but also reduces operating costs in the healthcare sector by providing technical expertise, guidance, and support in technology installation and configuration. Dedicated R&D explores various advanced technological options for I4.0 adoption to continue meeting pharmaceutical business needs and contributes to operational and SC sustainability. For example, efficient allocation and utilization of funds, tracking market intelligence, developing new ways to produce certain medicines to cut down production costs, upgrading lab and computing facilities with emerging technologies, and so on, can provide long-term sustainability through the SC. Previously, Sood et al. [83] also suggested that a dedicated, committed, and strong R&D team may play a vital role in PSC for producing excess revenue during the product life cycle and improving overall operational excellence by ensuring that technology performance is at its best.

“Sourcing and application of smart and advanced equipment (T3)” is ranked as the fourth most crucial factor. In the current highly competitive market, firms must focus increasingly on new sources of competitive application of data-driven, intelligent, and advanced equipment for promoting sustainability and gaining an edge over existing competitors. In PSC, application and sourcing have replaced human visual inspection of packaging, caps, and vials; predictive equipment maintenance has reduced disturbances, hazards, and production downtime. Thus, intelligent, advanced equipment can increase production output, raise manufacturing safety, improve quality, increase agility, offer flexibility, and minimize waste, which results in a hyper-connected, well-controlled, digitized ecosystem in the pharmaceutical value chain. Saha et al. [73] have also emphasized the application of intelligent and advanced equipment for overcoming pharmaceutical logistics and manufacturing barriers.

Government policies and legislation also play a significant role in implementing I4.0 in the pharmaceutical industry in the emerging economy. The government can support I.40 implementation by making policies that support and help firms respond to the call for global technological changes and adopt new emerging technologies to improve systems efficiently and flexibly. Fiscal policies and regulations can transform the manufacturing systems of traditional factories into technologically advanced futuristic manufacturing facilities with enhanced efficiency, accountability, and social responsibility to achieve sustainability. As a result, “Auspicious government policies and legislation (O3)” ranked as the 5^th^ most significant CSF in this research. Sharma et al. [84] have also identified government legislation, policies, and regulatory bodies as important contributors that can facilitate the successful adoption of I4.0.

“Maintain proper collaborative communication among supply chain (I4)” is the sixth most important CSF, which can boost organizational performance through customer service enhancement, improved resource utilization, and cost reduction. It significantly affects operational performance and responsiveness in every phase of the PSC, which can significantly reduce the bullwhip effect. Collaborative communication encourages enhanced openness and information sharing, which promote efficient decision-making and improve operational performance. Improved information sharing contributes to increased interconnection, leading to higher responsiveness in removing learning barriers and promoting greater understanding and communication among SC partners to increase sustainability [85].

Among all the CSFs, “advanced database management systems (I2)” and “decentralized management and flexible operations (T6)” had the most negligible impact. However, these CSFs are still important since they can assist pharmaceutical industry managers in improving their information processing, data management, and operational performance to facilitate long-term industrial sustainability [86].

Many studies have been performed in recent days on I4.0 implementation in various industrial sectors. However, our study varies from previous studies on I4.0 deployment in various industries from diverse economic circumstances, and the study’s findings also differ in many ways from the previous studies. For instance, El Baz et al. [87] investigated how sustainability drivers can be included in adopting Industry 4.0 technologies. They concluded that management support is a key driver, whereas economic advantages are the preferred sustainability externalities. Mahdiraji et al. [88] evaluated the possible interventions for the unsettling of I4.0 technologies in the circular supply chains of the pharmaceutical sector. They discovered that digital technology, environmental rules, subsidies, and rewards would help them transition to a lean, resilient, agile, and sustainable supply chain. Liu et al. [21] identified and rated the obstacles to deploying blockchain technology for SC sustainability. They found inter-organizational, intra-organizational, technical, and external barriers to be the most crucial factor in blockchain technology adaption. Again, Moeuf et al. [89] highlighted crucial success elements of I4.0 in SMEs and found a lack of experience, short-term strategy attitude, and lack of training to be the most crucial factor in implementing I4.0. However, the findings of these studies are not 100 percent applicable to PSC. On the other hand, our study analyzes the CSFs to implement I4.0 in the pharmaceutical industry while facilitating the achievement of the three bottom lines of sustainability, which no other studies have done yet. The study findings suggest Sufficient investment in technological advancement (T1), Digitalized product monitoring and traceability (I5), and Dedicated and robust Research and Development (R&D) teams (O5) to be the three most important CSFs to implement I4.0. These findings are quite unique and hence can provide unique perspectives to the managers and policymakers of the pharmaceutical industry to implement I4.0 and promote sustainability.

### 4.1 Theoretical implications

The study offers several important theoretical contributions. This study proposes a structured probabilistic group decision-making framework to identify, evaluate, and rank the CSFs that significantly impact the implementation of I4.0 and promote sustainability using a Bayesian BWM framework. This is one of the first attempts that connects PSC with the MCDM approach, I4.0, and sustainability. The study can contribute to Improving the theoretical comprehension of the present state of the pharmaceutical industry and developing plans for implementing I4.0 to achieve sustainability objectives, advancing widespread connectivity, and encouraging collaboration to improve this sector’s operational efficiency. The study also offers an in-depth understanding of how adopting I4.0 can increase productivity and reduce economic risk, failure rate, and defect rate while limiting the sector’s unfavorable environmental effects. This study can act as a benchmark for future scholars for more extensive research from different perspectives, providing decision-makers with more relevant data about CSFs to implement i4.0 in various other manufacturing sectors.

### 4.2 Managerial & policy implications

The study offers several critical implications for the pharmaceutical industry’s managers, practitioners, and policymakers. This structured study informs the managers about the significant CSFs to achieve PSC sustainability and prioritizes the key CSFs hierarchically. The study can help managers decide which CSFs to focus on first, especially when the industry operates with limited resources. For instance, sufficient investment in technological advancement (T1) is ranked first in the hierarchical ranking. Hence, the managers should start the I4.0 implementation with this CSF. Technology investment benefits stakeholders by increasing physical and intangible technological improvements for the comprehensive transformation of the SC toward sustainability. Moreover, the increased integration will improve the organization’s sustainability outcomes by ensuring on-time product monitoring and traceability, maintaining proper collaborative communication among SC entities, and creating an advanced database management system. Successful implementation of I4.0 in the pharmaceutical sector can morph their production system into a computer-integrated cloud manufacturing system that will link all vendors and manufacturers in a common node, allowing the sharing of data, knowledge, and information among the PSC entities. Therefore, this research can enable managers to devise efficient plans to achieve the identified CSFs and contribute to enhanced decision-making, productivity, and manufacturing efficiency.

The study suggests that government bodies and policymakers need to formulate fiscal policies and regulations and offer financial incentives to the pharmaceutical industries to promote I4.0 integration in their SC. This will encourage industries in an emerging economy to adopt I4.0 technologies more enthusiastically. This way, the study findings can guide policymakers to formulate long-term strategies that can directly and positively impact the organization’s sustainable development.

### 4.3 Implication of achieving sustainable development goals (SDGs)

I4.0 technologies can promote economic, social, and environmental sustainability in the PSC. This study’s findings can help managers and policymakers to achieve several specific SDGs. For instance:

- Implementing I4.0 technologies, such as IoT, blockchain, BDA, robotics, Internet of services (IoS), virtual reality, digital twin, cloud manufacturing, artificial intelligence, etc., can reduce the workload on human workers, improve job safety and environment and promote economic growth. This way, the study can help to achieve SDG 1 (Reducing Poverty) and SDG 8 (Decent work and economic growth).
- Sufficient investment for technological advancement, dedicated and robust Research and Development (R&D) team, and sourcing and application of intelligent and advanced equipment are the top three CSFs identified in this study. These CSFs can aid in streamlining the manufacturing processes, promote innovations, and enhance productivity. This way, the study can help to achieve SDG 9 (Infrastructure, Industry, and Innovation).
- Implementing I4.0 decreases pollution, waste generation, and energy consumption while improving productivity, resource utilization, and operational safety. This way, the study can help to achieve SDG 3 (Good Health and Well-Being) and SDG 12 (Responsible Consumption and Production).

## 5. Conclusions

The pharmaceutical sector is currently undergoing significant changes, driven by the need to innovate and reduce costs while maintaining compliance with regulatory requirements. Thus, I4.0 has become an increasingly important tool for pharmaceutical companies to streamline their SCs and improve their operations. The PSC has been exposed as a vulnerable sector by the recent COVID-19 pandemic, which has prompted a push for improved resilience, and spurred the extensive application of predictive analytics, digital inventory management, and blockchain technologies. Therefore, the emergence of I4.0 is crucial in promoting sustainability in the pharmaceutical industry by facilitating improved efficiency, transparency, and collaboration throughout the SC, leading to reduced waste, minimized environmental impact, and responsible production and sourcing practices. However, the drivers or CSFs of I4.0 implementation in the pharmaceutical industry have not yet been explored in an emerging economy context, exposing a research gap.

This research proposed a Bayesian BWM methodology to investigate and assess CSFs. First, the substantial CSFs were identified through a comprehensive literature search, which the area experts later validated. Then, using the experts’ feedback, Bayesian BWM, a novel group decision-making approach, was utilized to prioritize the identified CSFs systematically. The findings indicate that "Sufficient investment in technological advancement (T1)", " Digitalized product monitoring and traceability (I5)", and " Dedicated and robust Research and Development (R&D) team (O5)" are the top three ranked CSFs for I4.0 deployment in the pharmaceutical industry.

The study’s contribution can be assessed from various perspectives. The theoretical significance of this study lies in integrating the Bayes theorem with the BWM technique to analyze the CSFs for I4.0 implementations in the pharmaceutical industry, which has never been attempted before. Moreover, this approach can be replicated and adopted in other sectors beyond the pharmaceutical industry. The study’s findings will assist the managers and practitioners in efficiently transforming their production process into a computer-integrated manufacturing system to improve decision-making, productivity, and efficiency by focusing on the evaluated CSFs. Thus, it will facilitate the development of innovative ways to streamline manufacturing processes and enhance operational sustainability.

Like other studies, this research also comprises a few limitations that may be addressed in future research initiatives. The research concentrates particularly on the context of emerging economies, which may limit the applicability of the study’s findings globally. Therefore, future research needs to investigate other economic perspectives to provide a more comprehensive outlook. Moreover, the framework presented in this study was developed entirely based on data collected from industry experts working in the pharmaceutical industry. Nevertheless, some judgmental bias may have crept into the results, leading to outcomes that do not precisely reflect reality. One potential way to reduce the impact of opinion bias in future similar studies would be to improve expert diversity by expanding the pool of participant experts from various relevant sectors. Again, this research focused merely on the 16 most significant CSFs. New significant CSFs may emerge in the future, and this research may need to be extended to encompass these newly identified factors. Additionally, this study sought to prioritize CSFs only. To delve deeper, the interrelationships between these CSFs should be explored as well. A fuzzy or grey-based total interpretive structural modeling (TISM) approach can be integrated with future research for this purpose.

## Supporting information

S1 File Supplementary materials file. (DOCX)
