# Supplementary material for: Assessing the critical success factors for implementing industry 4.0 in the pharmaceutical industry: Implications for supply chain sustainability in emerging economies
Source: PLoS One. 2023 Jun 15;18(6):e0287149. doi: 10.1371/journal.pone.0287149 (PMC10270361; doi:10.1371/journal.pone.0287149)
Supplement: S1 File — (DOCX) [file pone.0287149.s001.docx]

**Supplementary Materials (S1 file)**

**Appendix A**

**Questionnaire for Assessing Critical Success Factors for Implementing Industry 4.0 in Pharmaceutical Industry: A Sustainable Supply Chain Development Perspective**

***Q.1:*** *What position do you hold in the pharmaceutical industry?*

***Q.2:*** *How many years of expertise do you have in Bangladesh's pharmaceutical industry?*

***Q.3:*** *Please choose the key CSFs to adopt Industry 4.0 toward sustainable supply chain development in the pharmaceutical industry from the CSFs provided below. If a CSF does have a substantial impact, please choose "Yes"; otherwise, select "No". Additionally, you are welcome to provide any additional CSFs that you think are essential for adopting Industry 4.0 in Bangladesh's pharmaceutical manufacturing industry.*

| **CSFs** | **Put "Yes" for relevant & "No" for irrelevant** |
| --- | --- |
| Existent sustainable organizational strategies |  |
| Auspicious government policies and legislation |  |
| Sufficient investment in technological advancement |  |
| Establishment of a robust technical support team |  |
| Training programs for employee skill advancement |  |
| Integration of advanced database management system |  |
| Proper support from top management |  |
| Digitalized product monitoring and traceability |  |
| Incorporation of emerging sustainable technologies |  |
| Establishment of the computer-integrated cloud manufacturing system |  |
| Organizational culture |  |
| Secured and stable data communication network |  |
| Maintain proper collaborative communication among supply chain entities |  |
| Technical Capabilities |  |
| Decentralized management and flexible operations |  |
| Increased automation in material handling and inventory management |  |
| Sourcing and application of smart and advanced equipment |  |
|  | |
| **Please provide any suggested CSFs** | |
| 1. | |
| 2. | |
| 3. | |
| 4. | |

**Table A1:** Brief description of selected CSFs

| **Sl.** | **CSFs** | **How do the factors impact on successful implementation of Industry 4.0 in supply chain sustainability** |
| --- | --- | --- |
| 1 | Existent sustainable organizational strategies | Sustainable organizational strategies focus on leadership commitment, collaboration, data-driven decision-making, innovation, continuous improvement, and employee engagement to increase organizational resource utilization and ensure the effectiveness of sustainable development to adopt I4.0. |
| 2 | Auspicious government policies and legislation | The government can structure the policies and regulations in such a way that pharmaceutical industries are motivated proactively in fostering and accelerating I4.0 adoption |
| 3 | Sufficient investment in technological advancement | Implementing I4.0 hardware, software, and training requires significant upfront capital costs. Moreover, by investing in these technologies, pharmaceutical industries can improve supply chain efficiency, reduce costs, enhance customer satisfaction, and gain a competitive advantage. |
| 4 | Establishment of a robust technical support team | The robust technical support team facilitates maintenance, troubleshooting, upgrades, updates, and training to ensure that the I4.0 technologies perform optimally and give the organization maximum value. |
| 5 | Training programs for employee skill advancement | The training programs help build leadership, facilitate positive changes, increase confidence in decision-making, support innovation, and ensure compliance and general aptitude among the employees to maximize the benefits of I4.0 technologies and drive business success. |
| 6 | Integration of advanced database management system | Advanced database management systems are applied to reduce the bullwhip effect by enabling data usability, visualization, transparency, security, and functionality among the different stakeholder departments. |
| 7 | Proper support from top management | The top management provides organizational support by setting strategic direction, building a culture of sustainability, providing leadership, allocating resources, and monitoring performance to drive the adoption of sustainable practices and I4.0. |
| 8 | Incorporation of emerging sustainable technologies | Incorporating emerging technologies such as IoT, additive manufacturing, blockchain, and so on can help to enhance supply chain sustainability by increasing transparency and traceability, optimizing the manufacturing process, reducing waste, and reducing carbon emissions. |
| 9 | Establishment of the computer-integrated cloud manufacturing system | Computer-integrated Cloud manufacturing (CICM) optimizes resource utilization, increases collaboration and transparency, improves flexibility and agility, and reduces the environmental impact with real-time manufacturing process monitoring. |
| 10 | Dedicated and robust Research and Development (R&D) team | Robust research and development enhance efficient allocation and utilization of funds and tracking market intelligence to achieve long-term sustainability. |
| 11 | Secured and stable data communication network | A secured and stable data communication network can enhance supply chain visibility, allowing better collaboration between suppliers, manufacturers, and distributors. This can help reduce the risk of supply chain disruptions and enable the adoption of various sustainable practices. |
| 12 | Maintain proper collaborative communication among supply chain entities | Maintaining proper collaborative communication among supply chain enhance profit generation, resource utilization, and cost reduction. It aids in promoting greater understanding and communication among supply chain partners to increase sustainability. |
| 13 | Digitalized product monitoring and traceability | Digital transformation of product monitoring and traceability can improve the consumer experience, streamline processes, ensure product safety and quality, and reduce waste. Moreover, it can enhance supply chain transparency, enable real-time data analysis, ensure regulatory compliance, and modify established business models by increasing customer responsiveness throughout the value chain entities. |
| 14 | Decentralized management and flexible operations | Flexible operations and decentralized management have high reconfigurability, expanding the importance of I4.0 technologies in large-scale distributed systems. |
| 15 | Sourcing and application of smart and advanced equipment | The new generation smart manufacturing equipment can overcome the inertia of conventional industrial infrastructure, operations, and regulations to reduce disturbances, hazards, waste, and production downtime, increase energy efficiency, enhance quality control, enable real-time monitoring and optimize inventory management. |
| 16 | Increased automation in material handling and inventory management | Automated material handling systems can prevent accidents, enhance operational efficiency, reduce labor expenses, increase workplace safety, and improve regulatory compliance. Furthermore, it enables the reduction of excess inventory, reduces waste due to product recalls, and improves consistency in the inventory. |

**Appendix B: Bayesian BWM**

**Table B1:** Recent studies using Bayesian BWM

| **Objectives** | **Area of research** | **Implemented Tool** | **Source** |
| --- | --- | --- | --- |
| To prioritize and best strategy searching for integrating suitability-feasibility-acceptability (SFA) strategies. | Battery, electronics, and automobile companies | SFA, Bayesian BWM,  MARCOS | [1] |
| To determine the criteria and sub-criteria weights for evaluating hospitals' preparation for disasters. | Hospital/ Healthcare industry | Bayesian BWM, VIKOR, and TOPSIS | [2] |
| Evaluating appropriate steps to secure employee safety, raw material availability, and customer purchase orders during COVID-19. | Ready-Made Garments (RMG) | Bayesian BWM | [3] |
| Identification and ranking of various impact factors | The Norwegian oil and gas industry | Bayesian BWM | [4] |
| To determine the best performance indicator of 5G Base Stations | Telecommunication | Bayesian BWM, DQ-GRA | [5] |
| Evaluating best groups and displaying influential interconnection | Sports and tourism  industry | Bayesian BWM, Rough DEMATEL | [6] |
| To prioritize CSFs for sustainable lean manufacturing | Furniture industry | Bayesian BWM | [7] |

**Appendix C: Best to Others vectors**

**Table C1:** Best to Others vector for the main cluster

| ***Experts*** | ***Most significant cluster*** | ***C1*** | ***C2*** | ***C3*** |
| --- | --- | --- | --- | --- |
| Expert 1 | C1. Organization and government-related cluster | 1 | 9 | 6 |
| Expert 2 | C1. Organization and government-related cluster | 1 | 9 | 5 |
| Expert 3 | C3. Information and network-related cluster | 9 | 2 | 1 |
| Expert 4 | C2. Technology and innovation-related cluster | 4 | 1 | 9 |
| Expert 5 | C1. Organization and government-related cluster | 1 | 3 | 9 |
| Expert 6 | C2. Technology and innovation-related cluster | 9 | 1 | 3 |
| Expert 7 | C2. Technology and innovation-related cluster | 9 | 1 | 2 |
| Expert 8 | C1. Organization and government-related cluster | 1 | 6 | 9 |
| Expert 9 | C3. Information and network-related cluster | 9 | 5 | 1 |
| Expert 10 | C2. Technology and innovation-related cluster | 5 | 1 | 9 |
| Expert 11 | C3. Information and network-related cluster | 7 | 9 | 1 |
| Expert 12 | C1. Organization and government-related cluster | 1 | 7 | 9 |

**Table C2:** Best to Others vector for Organizational and governmental-related sub-cluster

| ***Experts*** | ***Most significant-Organizational and governmental-related*** | ***O1*** | ***O2*** | ***O3*** | ***O4*** | ***O5*** |
| --- | --- | --- | --- | --- | --- | --- |
| Expert 1 | O5. Dedicated and robust Research and Development (R&D) team | 3 | 5 | 7 | 9 | 1 |
| Expert 2 | O1. Establishment of a robust technical support team | 1 | 9 | 5 | 2 | 7 |
| Expert 3 | O5. Dedicated and robust Research and Development (R&D) team | 9 | 7 | 6 | 3 | 1 |
| Expert 4 | O5. Dedicated and robust Research and Development (R&D) team | 9 | 8 | 2 | 6 | 1 |
| Expert 5 | O1. Establishment of a robust technical support team | 1 | 9 | 5 | 8 | 7 |
| Expert 6 | O4. Proper support from top management | 4 | 3 | 8 | 1 | 9 |
| Expert 7 | O5. Dedicated and robust Research and Development (R&D) team | 9 | 5 | 6 | 8 | 1 |
| Expert 8 | O2. Existent sustainable organizational strategies | 6 | 1 | 7 | 6 | 9 |
| Expert 9 | O2. Existent sustainable organizational strategies | 7 | 1 | 8 | 3 | 9 |
| Expert 10 | O2. Existent sustainable organizational strategies | 9 | 1 | 6 | 2 | 3 |
| Expert 11 | O5. Dedicated and robust Research and Development (R&D) team | 9 | 5 | 2 | 4 | 1 |
| Expert 12 | O1. Establishment of a robust technical support team | 1 | 9 | 3 | 6 | 4 |

**Table C3:** Best to Others vector for technology and innovation related cluster

| ***Experts*** | ***Most significant-Supply chain, inventory, and resource management related*** | ***T1*** | ***T2*** | ***T3*** | ***T4*** | ***T5*** | ***T6*** |
| --- | --- | --- | --- | --- | --- | --- | --- |
| Expert 1 | T1. Sufficient investment in technological advancement | 1 | 5 | 4 | 2 | 9 | 7 |
| Expert 2 | T3. Sourcing and application of smart and advanced equipment | 3 | 4 | 1 | 5 | 8 | 9 |
| Expert 3 | T6. Decentralized management and flexible operations | 4 | 9 | 2 | 6 | 8 | 1 |
| Expert 4 | T2. Training programs for employee skill advancement | 4 | 1 | 2 | 6 | 8 | 9 |
| Expert 5 | T2. Training programs for employee skill advancement | 8 | 1 | 7 | 5 | 6 | 9 |
| Expert 6 | T5. Increased automation in material handling and inventory management | 3 | 8 | 6 | 5 | 1 | 9 |
| Expert 7 | T3. Sourcing and application of smart and advanced equipment | 2 | 9 | 1 | 8 | 7 | 8 |
| Expert 8 | T2. Training programs for employee skill advancement | 7 | 1 | 5 | 6 | 9 | 8 |
| Expert 9 | T4. Incorporation of emerging sustainable technologies | 6 | 2 | 7 | 1 | 5 | 9 |
| Expert 10 | T3. Sourcing and application of smart and advanced equipment | 7 | 5 | 1 | 2 | 9 | 8 |
| Expert 11 | T1. Sufficient investment in technological advancement | 1 | 5 | 3 | 4 | 7 | 9 |
| Expert 12 | T1. Sufficient investment in technological advancement | 1 | 7 | 2 | 6 | 3 | 9 |

**Table C4:** Best to Others vector for information and network-related cluster

| ***Experts*** | ***Most significant-Performance and Technological related*** | ***I1*** | ***I2*** | ***I3*** | ***I4*** | ***I5*** |
| --- | --- | --- | --- | --- | --- | --- |
| Expert 1 | I2. Integration of advanced database management system | 4 | 1 | 9 | 2 | 7 |
| Expert 2 | I4. Maintain proper collaborative communication among supply chain entities | 9 | 3 | 7 | 1 | 4 |
| Expert 3 | I3. Establishment of the computer-integrated cloud manufacturing system | 7 | 2 | 1 | 9 | 8 |
| Expert 4 | I5. Digitalized product monitoring and traceability | 8 | 9 | 6 | 2 | 1 |
| Expert 5 | I5. Digitalized product monitoring and traceability | 8 | 9 | 6 | 3 | 1 |
| Expert 6 | I4. Maintain proper collaborative communication among supply chain entities | 6 | 8 | 7 | 1 | 9 |
| Expert 7 | I5. Digitalized product monitoring and traceability | 7 | 6 | 9 | 8 | 1 |
| Expert 8 | I3. Establishment of the computer-integrated cloud manufacturing system | 5 | 2 | 1 | 9 | 6 |
| Expert 9 | I5. Digitalized product monitoring and traceability | 7 | 9 | 8 | 6 | 1 |
| Expert 10 | I1. Secured and stable data communication network | 1 | 9 | 7 | 3 | 2 |
| Expert 11 | I4. Maintain proper collaborative communication among supply chain entities | 8 | 9 | 5 | 1 | 2 |
| Expert 12 | I3. Establishment of the computer-integrated cloud manufacturing system | 7 | 2 | 1 | 9 | 3 |

**Appendix D: Others to Worst Vectors**

**Table D1:** Others to Worst vector for the main cluster

| ***Experts*** | ***Least significant criteria*** | ***C1*** | ***C2*** | ***C3*** |
| --- | --- | --- | --- | --- |
| Expert 1 | C2. Technology and innovation-related cluster | 9 | 1 | 3 |
| Expert 2 | C2. Technology and innovation-related cluster | 9 | 1 | 7 |
| Expert 3 | C1. Organization and government-related cluster | 1 | 8 | 9 |
| Expert 4 | C3. Information and network-related cluster | 3 | 9 | 1 |
| Expert 5 | C3. Information and network-related cluster | 9 | 4 | 1 |
| Expert 6 | C1. Organization and government-related cluster | 1 | 9 | 4 |
| Expert 7 | C1. Organization and government-related cluster | 1 | 9 | 8 |
| Expert 8 | C3. Information and network-related cluster | 9 | 7 | 1 |
| Expert 9 | C1. Organization and government-related cluster | 1 | 2 | 9 |
| Expert 10 | C3. Information and network-related cluster | 3 | 9 | 1 |
| Expert 11 | C2. Technology and innovation-related cluster | 4 | 1 | 9 |
| Expert 12 | C3. Organizational and governmental | 9 | 5 | 1 |

**Table D2:** Others to Worst vector for organization and government-related cluster

| ***Experts*** | ***Least significant-Organizational and governmental-related*** | ***O1*** | ***O2*** | ***O3*** | ***O4*** | ***O5*** |
| --- | --- | --- | --- | --- | --- | --- |
| Expert 1 | O4. Proper support from top management | 7 | 6 | 4 | 1 | 9 |
| Expert 2 | O2. Existent sustainable organizational strategies | 9 | 1 | 6 | 7 | 8 |
| Expert 3 | O1. Establishment of a robust technical support team | 1 | 6 | 8 | 2 | 9 |
| Expert 4 | O1. Establishment of a robust technical support team | 1 | 8 | 7 | 5 | 9 |
| Expert 5 | O2. Existent sustainable organizational strategies | 9 | 1 | 3 | 5 | 6 |
| Expert 6 | O4. Proper support from top management | 3 | 5 | 9 | 1 | 8 |
| Expert 7 | O1. Establishment of a robust technical support team | 1 | 8 | 4 | 7 | 9 |
| Expert 8 | O5. Dedicated and robust Research and Development (R&D) team | 6 | 9 | 7 | 8 | 1 |
| Expert 9 | O5. Dedicated and robust Research and Development (R&D) team | 2 | 9 | 3 | 5 | 1 |
| Expert 10 | O1. Establishment of a robust technical support team | 1 | 8 | 9 | 7 | 4 |
| Expert 11 | O1. Establishment of a robust technical support team | 1 | 3 | 4 | 2 | 9 |
| Expert 12 | O2. Existent sustainable organizational strategies | 9 | 1 | 7 | 2 | 6 |

**Table D3:** Others to Worst vector for technology and innovation-related cluster

| ***Experts*** | ***Technology and innovation-related*** | ***T1*** | ***T2*** | ***T3*** | ***T4*** | ***T5*** | ***T6*** |
| --- | --- | --- | --- | --- | --- | --- | --- |
| Expert 1 | T5. Increased automation in material handling and inventory management | 9 | 7 | 8 | 6 | 1 | 3 |
| Expert 2 | T6. Decentralized management and flexible operations | 7 | 6 | 9 | 4 | 8 | 1 |
| Expert 3 | T2. Training programs for employee skill advancement | 8 | 1 | 3 | 6 | 7 | 9 |
| Expert 4 | T6. Decentralized management and flexible operations | 8 | 9 | 7 | 2 | 6 | 1 |
| Expert 5 | T6. Decentralized management and flexible operations | 4 | 3 | 5 | 3 | 9 | 1 |
| Expert 6 | T6. Decentralized management and flexible operations | 9 | 8 | 2 | 5 | 7 | 1 |
| Expert 7 | T2. Training programs for employee skill advancement | 5 | 1 | 9 | 4 | 8 | 8 |
| Expert 8 | T5. Increased automation in material handling and inventory management | 7 | 9 | 6 | 5 | 1 | 3 |
| Expert 9 | T6. Decentralized management and flexible operations | 8 | 9 | 7 | 2 | 8 | 1 |
| Expert 10 | T5. Increased automation in material handling and inventory management | 4 | 2 | 9 | 8 | 1 | 3 |
| Expert 11 | T6. Decentralized management and flexible operations | 9 | 7 | 6 | 5 | 7 | 1 |
| Expert 12 | T6. Decentralized management and flexible operations | 9 | 7 | 2 | 5 | 8 | 1 |

**Table D4:** Others to Worst vector for Information and network related cluster

| ***Experts*** | ***Least significant- Information and network related*** | ***I1*** | ***I2*** | ***I3*** | ***I4*** | ***I5*** |
| --- | --- | --- | --- | --- | --- | --- |
| Expert 1 | I3. Establishment of the computer-integrated cloud manufacturing system | 5 | 9 | 1 | 7 | 3 |
| Expert 2 | I1. Secured and stable data communication network | 1 | 5 | 7 | 9 | 3 |
| Expert 3 | I4. Maintain proper collaborative communication among supply chain entities | 4 | 2 | 9 | 1 | 8 |
| Expert 4 | I2. Integration of advanced database management system | 8 | 1 | 7 | 6 | 9 |
| Expert 5 | I2. Integration of advanced database management system | 3 | 1 | 2 | 4 | 9 |
| Expert 6 | I5. Digitalized product monitoring and traceability | 5 | 3 | 6 | 9 | 1 |
| Expert 7 | I3. Establishment of the computer-integrated cloud manufacturing system | 6 | 7 | 1 | 5 | 9 |
| Expert 8 | I4. Maintain proper collaborative communication among supply chain entities | 7 | 6 | 9 | 1 | 8 |
| Expert 9 | I5. Digitalized product monitoring and traceability | 5 | 3 | 9 | 4 | 1 |
| Expert 10 | I2. Integration of advanced database management system | 9 | 1 | 3 | 8 | 5 |
| Expert 11 | I2. Integration of advanced database management system | 3 | 1 | 2 | 6 | 8 |
| Expert 12 | I4. Maintain proper collaborative communication among supply chain entities | 6 | 4 | 9 | 1 | 5 |

**Appendix E: Confidence level scores of clusters and sub-clusters**

**Table E1:** Major cluster level confidence score

| ***Criteria*** | ***C1*** | ***C2*** | ***C3*** |
| --- | --- | --- | --- |
| C1. Organization and government-related cluster | 0.0000 | 0.3375 | 0.5486 |
| C2. Technology and innovation-related cluster | 0.6625 | 0.0000 | 0.7054 |
| C3. Information and network-related cluster | 0.4514 | 0.2946 | 0.0000 |

**Table E2:** Organization and government-related CSFs confidence score

| ***Sub-criteria (CSFs)*** | ***S1*** | ***S2*** | ***S3*** | ***S4*** | ***S5*** |
| --- | --- | --- | --- | --- | --- |
| O1. Establishment of a robust technical support team | 0.0000 | 0.2457 | 0.1621 | 0.3013 | 0.0769 |
| O2. Existent sustainable organizational strategies | 0.7543 | 0.0000 | 0.3791 | 0.5666 | 0.2230 |
| O3. Auspicious government policies and legislation | 0.8379 | 0.6208 | 0.0000 | 0.6827 | 0.3230 |
| O4. Proper support from top management | 0.6987 | 0.4333 | 0.3173 | 0.0000 | 0.1778 |
| O5. Dedicated and robust Research and Development (R&D) team | 0.9231 | 0.7770 | 0.6769 | 0.8222 | 0.0000 |

**Table E3:** Technology and innovation-related CSFs confidence score

| ***Sub-criteria (CSFs)*** | ***O1*** | ***O2*** | ***O3*** | ***O4*** | ***O5*** | ***O6*** |
| --- | --- | --- | --- | --- | --- | --- |
| T1. Sufficient investment in technological advancement | 0.0000 | 0.9056 | 0.6516 | 0.9624 | 0.9853 | 0.9999 |
| T2. Training programs for employee skill advancement | 0.0944 | 0.0000 | 0.1777 | 0.6795 | 0.8152 | 0.9976 |
| T3. Sourcing and application of smart and advanced equipment | 0.3483 | 0.8223 | 0.0000 | 0.9152 | 0.9649 | 0.9999 |
| T4. Incorporation of emerging sustainable technologies | 0.0377 | 0.3205 | 0.0848 | 0.0000 | 0.6667 | 0.9928 |
| T5. Increased automation in material handling and inventory management | 0.0147 | 0.1848 | 0.0351 | 0.3333 | 0.0000 | 0.9786 |
| T6. Decentralized management and flexible operations | 0.0001 | 0.0024 | 0.0001 | 0.0072 | 0.0214 | 0.0000 |

**Table E4:** Information and network-related cluster CSFs confidence score

| ***Sub-criteria (CSFs)*** | ***I1*** | ***I2*** | ***I3*** | ***I4*** | ***I5*** |
| --- | --- | --- | --- | --- | --- |
| I1. Secured and stable data communication network | 0.0000 | 0.6433 | 0.3550 | 0.2363 | 0.0887 |
| I2. Integration of advanced database management system | 0.3567 | 0.0000 | 0.2319 | 0.1408 | 0.0451 |
| I3. Establishment of the computer-integrated cloud manufacturing system | 0.6450 | 0.7681 | 0.0000 | 0.3648 | 0.1618 |
| I4. Digitalized product monitoring and traceability | 0.7637 | 0.8592 | 0.6352 | 0.0000 | 0.2604 |
| I5. Maintain proper collaborative communication among supply chain entities | 0.9113 | 0.9549 | 0.8382 | 0.7396 | 0.0000 |

**References**

[1] Hashemkhani Zolfani S, Bazrafshan R, Ecer F, Karamaşa Ç. The suitability-feasibility-acceptability strategy integrated with Bayesian BWM-MARCOS methods to determine the optimal lithium battery plant located in South America. Mathematics. 2022 Jul 8;10(14):2401. <https://doi.org/10.3390/math10142401>

[2] Saner HS, Yucesan M, Gul M. A Bayesian BWM and VIKOR-based model for assessing hospital preparedness in the face of disasters. Natural hazards. 2022 Mar 1:1-33. <https://doi.org/10.1007/s11069-021-05108-7>

[3] Munim ZH, Balasubramaniyan S, Kouhizadeh M, Hossain NU. Assessing blockchain technology adoption in the Norwegian oil and gas industry using Bayesian Best Worst Method. Journal of Industrial Information Integration. 2022 Jul 1;28:100346. <https://doi.org/10.1016/j.jii.2022.100346>

[4] Munim ZH, Mohammadi M, Shakil MH, Ali SM. Assessing measures implemented by export-oriented RMG firms in an emerging economy during COVID-19. Computers & Industrial Engineering. 2022 Mar 1;165:107963. <https://doi.org/10.1016/j.cie.2022.107963>

[5] Liang M, Li W, Ji J, Zhou Z, Zhao Y, Zhao H, Guo S. Evaluating the Comprehensive Performance of 5G Base Station: A Hybrid MCDM Model Based on Bayesian Best-Worst Method and DQ-GRA Technique. Mathematical Problems in Engineering. 2022 Jan 31;2022:1-5. <https://doi.org/10.1155/2022/4038369>

[6] Yang JJ, Lo HW, Chao CS, Shen CC, Yang CC. Establishing a sustainable sports tourism evaluation framework with a hybrid multi-criteria decision-making model to explore potential sports tourism attractions in Taiwan. Sustainability. 2020 Feb 23;12(4):1673. <https://doi.org/10.3390/su12041673>

[7] Debnath B, Shakur MS, Bari AM, Karmaker CL. A Bayesian Best–Worst approach for assessing the critical success factors in sustainable lean manufacturing. Decision Analytics Journal. 2023 Mar 1;6:100157. <https://doi.org/10.1016/j.dajour.2022.100157>
